# Herb pair of Huangqi‐Danggui exerts anti‐tumor immunity to breast cancer by upregulating *PIK3R1*

**DOI:** 10.1002/ame2.12434

**Published:** 2024-06-11

**Authors:** Hai‐Xin Liu, Li Lian, Li‐Li Hou, Cai‐Xia Liu, Jin‐Hong Ren, Yuan‐Biao Qiao, Shi‐Yuan Wen, Qing‐Shan Li

**Affiliations:** ^1^ College of Traditional Chinese Medicine and Food Engineering Shanxi University of Chinese Medicine Taiyuan China; ^2^ Shanxi Key Laboratory of Innovative Drug for the Treatment of Serious Diseases Basing on the Chronic Inflammation, College of Traditional Chinese Medicine and Food Engineering Shanxi University of Chinese Medicine Taiyuan China; ^3^ College of Basic Medical Sciences Shanxi Medical University Taiyuan China

**Keywords:** anti‐tumor, Danggui, Huangqi, immunity

## Abstract

**Background:**

According to traditional Chinese medicine (TCM), drugs supplementing the vital energy, Qi, can eliminate tumors by restoring host immunity. The objective of this study is to investigate the underlying immune mechanisms of anti‐tumor activity associated with Qi‐supplementing herbs, specifically the paired use of Huangqi and Danggui.

**Methods:**

Analysis of compatibility regularity was conducted to screen the combination of Qi‐supplementing TCMs. Using the MTT assay and a transplanted tumor mice model, the anti‐tumor effects of combination TCMs were investigated in vitro and in vivo. High content analysis and flow cytometry were then used to evaluate cellular immunity, followed by network pharmacology and molecular docking to dissect the significant active compounds and potential mechanisms. Finally, the anti‐tumor activity and the mechanism of the active ingredients were verified by molecular experiments.

**Results:**

There is an optimal combination of Huangqi and Danggui that, administered as an aqueous extract, can activate immunity to suppress tumor and is more effective than each drug on its own in vitro and in vivo. Based on network pharmacology analysis, PIK3R1 is the core target for the anti‐tumor immunity activity of combined Huangqi and Danggui. Molecular docking analysis shows 6 components of the combined Danggui and Huangqi extract (quercetin, jaranol, isorhamnetin, kaempferol, calycosin, and suchilactone) that bind to PIK3R1. Jaranol is the most important component against breast cancer. The suchilactone/jaranol combination and, especially, the suchilactone/kaempferol combination are key for immunity enhancement and the anti‐tumor effects of the extract.

**Conclusions:**

The combination of Huangqi and Danggui can activate immunity to suppress breast cancer and is more effective than the individual drugs alone.

## Graphical abstract

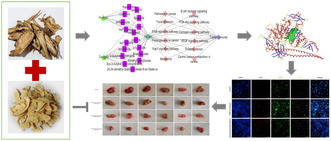

## 1 INTRODUCTION

Breast cancer is the most prevalent tumor impacting women's health. Currently, early detection of breast cancer can be more effectively treated through surgical removal. However, the low cure rate associated with highly metastatic and drug‐resistant breast cancer significantly impairs the quality of life for women. The heterogeneity of this disease poses a substantial challenge for therapeutic management.^1^ Nevertheless, recent progress in molecular biology and immunology has enabled the development of highly targeted therapies for various types of breast cancer.^2^ The clinical application of immune checkpoint inhibitors for triple‐negative breast cancer has shown mixed results, with only a minority of patients achieving a durable response.^3^ Over recent years, immunotherapy has become increasingly important in the treatment of tumors, as it activates the immune cells' ability to eliminate cancer cells.^4^, ^5^ Despite the improved therapeutic outcomes associated with tumor immunotherapy, many malignant tumors still manage to evade the immune system's surveillance through various mechanisms.^6^, ^7^ Given that immunotherapy alone is not sufficient to eradicate tumors or to ensure long‐term antitumor effects, there is a growing trend towards combining it with other tumor treatment methods that can stimulate an immune response. In this context, the development of chemotherapy drugs that target breast cancer by enhancing the body's immune system represents a promising avenue for future therapeutic strategies.

Numerous studies have demonstrated that Traditional Chinese Medicine (TCM) has a significant impact on tumor suppression, reduces drug resistance, and enhances the immune response while mitigating adverse reactions.^8^, ^9^, ^10^ In recent years, a breakthrough in TCM has been made through targeting immune checkpoints, such as PD‐1 and PD‐L1, thus offering a novel approach in the treatment of various malignant tumors.^11^, ^12^ TCM posits that Qi, the vital energy, is the driving force behind all physiological functions of the human body. It is generated by the combination of the essence of water and grain with the natural atmosphere that is inhaled. The Yellow Emperor's Classic of Internal Medicine suggests that a body filled with positive energy will repel evil energy, preventing illness. The concept of tumor elimination through the restoration of host immunity aligns with the TCM principle of “nourishing the positive accumulation and allowing cancer to be eliminated by itself” or “strengthening vital Qi to treat cancer”.^13^, ^14^ Therefore, Qi‐supplementing drugs within TCM have the potential to overcome tumors by bolstering the immune system. By enhancing the body's natural defenses, these treatments can create an environment that is less conducive to tumor growth and more capable of fighting cancer. This approach not only complements modern oncological therapies but may also pave the way for innovative cancer treatments that integrate the wisdom of TCM with contemporary medical science.

*Astragalus membranaceus* (Fisch.) Bunge, commonly referred to as Huangqi in Traditional Chinese Medicine (TCM), is the root of either *A. membranaceus* Bge. var. mongholicus (Bge.) Hsiao or *A. membranaceus* (Fisch.) Bge. Huangqi is renowned for its ability to “invigorate Qi and raise Yang,” as documented in TCM, and its processed forms are listed in the Chinese Pharmacopeia for treating “Qi deficiency” syndrome.^15^ For millennia, Huangqi has been esteemed as a royal medicine in TCM herbal composite formulae (Fufang) by practitioners. Numerous studies have indicated that Huangqi is beneficial for patients with deficiencies in ‘Qi’ and ‘blood’, playing a role in anti‐tumor activity, antioxidant effects, and alleviating complications associated with cardiovascular diseases in clinical practice.^16^ Moreover, the ‘Qi’ and ‘blood’‐supplementing effects of Huangqi are closely linked to enhancement of the host's immune system.^17^, ^18^ In the context of network pharmacology, Huangqi is considered the central drug in screening for compatible TCMs within herbal composite formulae, potentially increasing their efficacy in anti‐tumor treatment. *Angelica sinensis* (Oliv.) Diels, known as Danggui in TCM, can be combined with other TCM ingredients to counteract various antigens.^19^ Its primary components have been shown to stimulate the immune system and exhibit immuno‐stimulatory effects.^20^ By integrating Huangqi with other TCM ingredients like Danggui, the potential for developing effective anti‐tumor therapies that leverage the immune system is promising. This approach not only draws on the ancient wisdom of TCM but also incorporates modern scientific understanding to create a comprehensive and synergistic treatment strategy.

The potential synergistic effects of the herbal combination of Huangqi and Danggui on anti‐tumor immunity and their comparative efficacy against single herb treatments remain uncertain. The objective of this study is to investigate the underlying immune mechanisms of anti‐tumor activity associated with Qi‐supplementing herbs, specifically the paired use of Huangqi and Danggui. The findings aim to establish a foundation for understanding the role of TCM herbal pairs in enhancing anti‐tumor immunity.

## 2 METHODS

### 2.1 Analysis of compatibility patterns in TCM prescriptions

Traditional Chinese Medicine (TCM) prescriptions that are categorized as treatments for ‘Yiqi’ (qi deficiency) or ‘Buqi’ (lack of qi) were sourced from the Pharmacopeia of the People's Republic of China (2020 edition), Yaoyuan web, and relevant literature.^21^ These resources were utilized to compile a comprehensive database of Qi‐supplementing herbs. Subsequently, data mining techniques were applied to identify high‐frequency TCM herbs and pivotal combinations within the TCM framework, using the TCMISS.

### 2.2 Identification of Huangqi and Danggui ingredients

The chemical ingredients of Huangqi and Danggui were collected by the Traditional Chinese Medicine Systems Pharmacology Database and Analysis Platform (TCMSP, http://tcmspw.com/tcmsp.php) and the Traditional Chinese Medicine Integrated Database (TCMID, http://119.3.41.228:8000/tcmid/search/). We used the following filters: oral bioavailability (OB) greater than or equal to 30%, and drug‐likeness (DL) greater than or equal to 0.18. Furthermore, the PubChem (http://119.3.41.228:8000/tcmid/search/) was used for obtaining the Canonical simplified molecular input line entry specification (SMILES) information of these ingredients.

### 2.3 Screening ingredient targets for Huangqi and Danggui

All the targets related to the bioactive ingredient sof Huangqi and Danggui were obtained from the Swiss Target Prediction webtool (http://www.swisstargetprediction.ch/). Swiss Target Prediction is a network server that predicts potential drug targets by querying ingredients through reverse pharmacophore matching of internal pharmacophore model database entries. Only targets with a norm probability (in Swiss Target Prediction) higher than 0.60 would be selected, to ensure reliability of prediction.^22^, ^23^

### 2.4 Disease‐associated targets

We uploaded ‘tumor immunity’ as a search term to the DisGeNET (http://www.disgenet.org/search), Genecards (Score greater than or equal to 20) (https://www.genecards.org/) and NCBI Gene (https://www.ncbi.nlm.nih.gov/gene/) databases.^22^, ^24^, ^25^ These three databases reveal the relationship between targets and diseases from different perspectives.

### 2.5 Protein–protein interaction (PPI) network

To demonstrate the interaction between target proteins, the search terms ‘tumor‐related immunity’ and ‘ingredient target proteins’ were uploaded to STRING (http://string‐db.org) to obtain PPI information. Only ‘Homo sapiens’ proteins were screened out based on their confidence score greater than 0.7. Then a PPI network was constructed using Cytoscape 3.2.1 software.

### 2.6 Central network evaluation

To evaluate the intersection, the CytoNCA plug‐in in Cytoscape was used. The data were filtered based on three centrality measures provided by CytoNCA, including degree centrality (DC), closeness centrality (CC), and betweenness centrality (BC). Firstly, ‘DC greater than or equal to 2× median DC’ screening criteria are used for preliminary data processing. Then, secondary screening was conducted using the screening criteria of ‘DC, BC, and CC greater than or equal to the median’ as the core targets.

### 2.7 Enrichment analysis

We used the DAVID webtool (https://david.nicifcrf.gov/) for GO enrichment analysis with the *p* value <0.05 and KEGG pathway enrichment analysis with the *p* value <0.05 and *q* value <0.05.

### 2.8 Molecular docking

The protein conformations were retrieved from the Protein Data Bank (PDB) database, which can be accessed at https://www.rcsb.org/. The criteria for protein selection were as follows: (1) protein structures were obtained based on X‐ray crystallography; (2) the crystal resolution of the proteins was 2.8 Å or better; (3) priority was given to protein structures that have been reported in molecular docking literature; (4) the proteins of interest were those derived from *Homo sapiens*. Subsequently, molecular docking calculations were carried out using the software SYBYL‐X 2.0.

### 2.9 Extraction method

Six groups of combinations of Danggui and Huangqi in different proportions were prepared: Namely, the Danggui‐only group (72 g), the Huangqi‐only group (72 g), and the following Danggui/Huangqi combination groups: 1:1 group (36 g Danggui, 36 g Huangqi), 1:2 group (24 g Danggui, 48 g Huangqi), 1:3 group (18 g Danggui, 54 g Huangqi), 1:4 groups (14.4 g Danggui, 57.6 g Huangqi), 1:5 groups (12 g Danggui, 60 g Huangqi). The herbs were heated and infused by water for 2 hours, and then filtered. The herbs were extracted three times in this way. Finally, the filtrates were combined, concentrated, and freeze‐dried to a powder. The powder was stored for subsequent use in cell and animal experiments.

### 2.10 High content evaluation of co‐culture cells

Mouse spleen was dissected and then triturated to obtain single cells. The murine spleen cells were suspended in RPMI1640 (Gibco, Thermo Fisher Scientific, Waltham, MA, USA) medium suspended with 10% fetal bovine serum (FBS, Cell Max), 50 mg/mL of streptomycin and 50 U/mL of penicillin G (Boster, Pleasanton, CA, USA) and cultured at 37°C, 5% CO_2_ to pre‐grow for 24 h. Then, 4T1 cells (ATCC) were added for co‐culture with the murine spleen cells in 96 well plates (1:2, 4000 cells/well 4T1 cells, and 8000 cells/well murine spleen cells).

### 2.11 RT‐qPCR analysis

The co‐cultured cells, consisting of 4T1 cells and murine spleen cells in a 1:1 ratio, were treated with a panel of ingredients including quercetin, jaranol, isorhamnetin, kaempferol, calycosin, and suchilactone, each at a concentration of 1 μmol/L for a period of 24 h. Following the treatment, total RNA was extracted from the 4T1 cells using Trizol reagent. The subsequent experimental procedures were carried out in accordance with the methods described in our previous study.^26^ The sequences of primers used for RT‐qPCR are as follows: GAPDH: F: 5′‐GGCACAGTCAAGGCTGAGAATG‐3′, R: 5′‐ATGGTGGTGAAGACGCCAGTA‐3′; PIK3R1: F: 5′‐AAACAAAGCGGAGAACCTATTG‐3′, R: 5′‐TAATGACGCAATGCTTGACTTC‐3′.

### 2.12 MTT assay

The co‐cultured cells (4T1 cells: murine spleen cells = 1:1) explanted into 96 wells were treated with the same ingredients (quercetin, jaranol, isorhamnetin, kaempferol, calycosin, or suchilactone) alone at 1 μmol/L, or in combination (1 μmol/L), or with combined Danggui and Huangqi (100 μg/mL) for 24 h. Then, 3‐(4,5‐dimethylthiazol‐2‐yl)‐2,5‐diphenyltetrazolium bromide (MTT, Solarbio) was added at 0.1 mg/mL final concentration to each well for 4 h. The following experimental process was completed using our previous experimental method.^27^

### 2.13 Enzyme‐linked immunosorbent assays (ELISA)

Following the treatment with individual ingredients (quercetin, jaranol, isorhamnetin, kaempferol, calycosin, or suchilactone) at a concentration of 1 μmol/L, the combined ingredients at the same concentration, or the combination of Danggui and Huangqi at a concentration of 100 μg/mL for 24 h, the level of interferon‐gamma (IFN‐γ) in the supernatant of co‐cultured cells (with a ratio of 4T1 cells to murine spleen cells of 1:1) was determined using an ELISA kit (AD3421Mo, Andy Gene, Beijing, China). The procedure involved adding the cell supernatant to the ELISA plate and incubating it at 37°C for 30 min. Afterwards, the plate was washed five times for 0.5 min with the buffer to remove unbound substances and then incubated with the HRP‐Conjugate reagent for an additional 30 min. Subsequently, Chromogen Solution A and Chromogen Solution B were added to the wells and incubated at 37°C in the dark for 5 min to facilitate color development. The reaction was stopped by adding the termination buffer. Finally, the optical density (OD) was measured at a wavelength of 450 nm using a microplate reader. To determine the concentration of IFN‐γ in the samples, the OD values obtained were compared to a standard curve generated using known concentrations of IFN‐γ. This allowed the calculation of the IFN‐γ concentration in the samples based on the standard concentrations and the measured OD values.

### 2.14 Animal model

The tumor animal model was established using 4T1 cells xenografted in female BALB/c mice (Sibeifu Beijing biology technology limited company, SCXK 2019–0010). 4T1 cells (1 × 10^5^) were inoculated onto the right armpit of immunocompetent female BALB/C mice. These mice were separated into 6 groups (model group, low‐dose of Danggui and Huangqi group, medium‐dose of Danggui and Huangqi group, high‐dose of Danggui and Huangqi group, Danggui group, and Huangqi group) with an even distribution of tumor volumes (n = 6/group). A week after the xenograft tumor was established, the mice were treated with the drugs. All the animal models were treated by intragastric administration (i.g.) every day for 20 days, and the dosage of drugs used in each group was as follows: (1) low‐dose of Danggui and Huangqi group, Danggui (0.4 g/kg) and Huangqi (1.2 g/kg), (2) medium‐dose of Danggui and Huangqi group, Danggui (0.8 g/kg) and Huangqi (2.4 g/kg), (3) high‐dose of Danggui and Huangqi group, Danggui (1.2 g/kg) and Huangqi (3.6 g/kg), (4) Danggui group, Danggui (1.2 g/kg), (5) Huangqi group, Huangqi (3.6 g/kg), and (6) model group, normal saline (100 mL) as a mock agent.

The mice body weights were measured every day, and tumor diameters were measured in two dimensions every day using a caliper. The tumor volume was calculated according to the following formula: volume (mm^3^) = (width)^2^ × (length) × 1/2. After the mice were killed by cervical dislocation, the tumor was stripped and weighed. Additionally, the heart, liver, spleen, lungs, and kidneys were individually weighed and harvested for subsequent Hematoxylin and Eosin (H&E) staining.

### 2.15 Flow cytometric analysis of splenic immune cells

Following the euthanasia of the mice via cervical dislocation, their spleens were carefully extracted and processed into a single‐cell suspension. The cells were then resuspended in staining buffer and allowed to incubate with fluorophore‐conjugated anti‐mouse monoclonal antibodies for a period of 30 min at room temperature in a dark environment to prevent photobleaching and ensure optimal staining conditions. The fluorophore‐conjugated anti‐mouse monoclonal antibodies (mAbs, BioLegend) are as follows: FITC anti‐mouse/human CD45R/B220 Antibody (103205), PE anti‐mouse CD3 Antibody (100205), FITC anti‐mouse CD8a Antibody (100705), APC/Cyanine7 anti‐mouse CD4 (100525), PE anti‐mouse/human CD11b Antibody (100525), APC anti‐mouse CD11c Antibody (101207), FITC anti‐mouse CD80 Antibody (104705), APC anti‐mouse IFN‐γ Antibody (505809), FITC anti‐mouse H‐2Kb Antibody (116505), Pacific Blue™ anti‐mouse I‐A/I‐E Antibody (107619), and FITC anti‐mouse NK‐1.1 Antibody (108705). Finally, the cells were resuspended in PBS for flow cytometry (BD LSR II) and the data obtained were analyzed using FlowJo and GraphPad Prism 5 software.

## 3 RESULTS

### 3.1 A combination of Huangqi and Danggui is considered the optimal herbal pairing for the purpose of supplementing Qi in TCM

In order to analyze the frequency of individual herbs used in TCM prescriptions aimed at supplementing Qi, a total of 238 Qi‐supplementing prescriptions were collected from the Chinese Pharmacopeia (CHP, 2020 edition), Yaoyuan web, and relevant literature, with duplicates excluded. These prescriptions comprised 387 different TCM herbs. Through frequency analysis, 45 herbs that are commonly used for supplementing Qi were identified (with a frequency of 10 or higher, as detailed in Table 1). Among these, the top three most frequently used herbs were Huangqi (*Astragalus membranaceus*), Dangshen (*radix codonopsis*), and Danggui (*Angelica sinensis*).

**TABLE 1:** Frequency of herbs in the Chinese traditional patent medicine for supplementing Qi (frequency ≥ 10).

No.	Name	Frequency	No.	Name	Frequency
1	Huangqi	120	24	Zhihuangqi	20
2	Dangshen	68	25	Dazao	19
3	Danggui	63	26	Hongshen	19
4	Danshen	55	27	Sanqi	18
5	Renshen	52	28	Chishao	16
6	Fuling	50	29	Guizhi	14
7	Baizhu	46	30	Rougui	14
8	Chuangxiong	45	31	Huangjing	14
9	Gancao	41	32	Niuxi	13
10	Maidong	39	33	Bingpian	13
11	Baishao	36	34	Tusizi	13
12	Shoudihuang	35	35	Heshouwu	12
13	Wuweizi	35	36	Huanglian	12
14	Gouqizi	29	37	Ejiao	12
15	Shanyao	28	38	Muxiang	12
16	Yinyanghuo	26	39	Jixueteng	11
17	Gegen	25	40	Taoren	11
18	Dihuang	25	41	Tianhuangfen	11
19	Chenpi	25	42	Dilong	11
20	Zhigancao	23	43	Yuanzhi	11
21	Shanzha	22	44	Lurong	10
22	Zhiheshouwu	21	45	Suangzaoren	10
23	Honghua	20	46	Dahuang	10

In the subsequent analysis, the compatibility patterns within TCM prescriptions for supplementing Qi were statistically examined. By setting the support threshold to a minimum of 20 and the confidence to a minimum of 0.9 within the TCMISS, a mining of compatibility patterns within the prescriptions was conducted. This analysis yielded 18 key herbal combinations that are effective for supplementing Qi, as presented in Table 2. Prominently, the most frequently occurring combination was found to be Huangqi and Danggui.

**TABLE 2:** Core combinations with high frequency mined from supplementing Qi formulae (support number: 20, confidence ≥0.9).

NO.	Core combination	Frequency
1	Huangqi, Danggui	35
2	Danshen, Huangqi	33
3	Dangshen, Huangqi	32
4	Renshen, Huangqi	29
5	Chuanxiong, Danggui	28
6	Chuanxiong, Huangqi	28
7	Dangshen, Danggui	27
8	Dangshen, Baizhu	25
9	Baizhu, Danggui	23
10	Huangqi, Baizhu	23
11	Baizhu, Fuling	23
12	Gegen, Huangqi	22
13	Huangqi, Gancao	22
14	Dangshen, Baishao	21
15	Dangshen, Fuling	21
16	Maidong, Huangqi	21
17	Shoudihuang, Danggui	20
18	Chuanxiong, Danshen	20

Further analysis was carried out using association rule mining for Qi‐supplementing TCM prescriptions, with the support set to a minimum of 15 and confidence to a minimum of 0.7 within TCMISS. The analysis revealed that when Huangqi is included in a prescription, there is approximately a 97% probability that Danggui is also present, as detailed in Table 3. This finding further substantiates the frequent use of this particular herb pairing in TCM practice. TCM prescriptions that contain both Huangqi and Danggui include Bu Zhong Yi Qi Wan, Yiqi Yangxue Oral Liquid, Gui Pi Wan, Bai Dian Feng Wan, Compound Huangqi Oral Liquid, and Qingfei Shibawei Wan.

**TABLE 3:** Association rule of supplementing Qi formulae (support number: 15, confidence ≥0.7).

NO.	Association rules	Confidence
1	Huangqi → Danggui	0.972222
2	Chuanxiong → Danggui	0.954545
3	Dangshen → Danggui	0.95
4	Baizhu → Danggui	0.944444
5	Chuanxiong, Huangqi → Danggui	0.944444
6	Chuanxiong → Huangqi	0.818182
7	Chuangxiong, Danggui → Huangqi	0.809524
8	Chuanxiong → Huangqi, Danggui	0.772727
9	Danggui → Huangqi	0.729167

**FIGURE 1:**
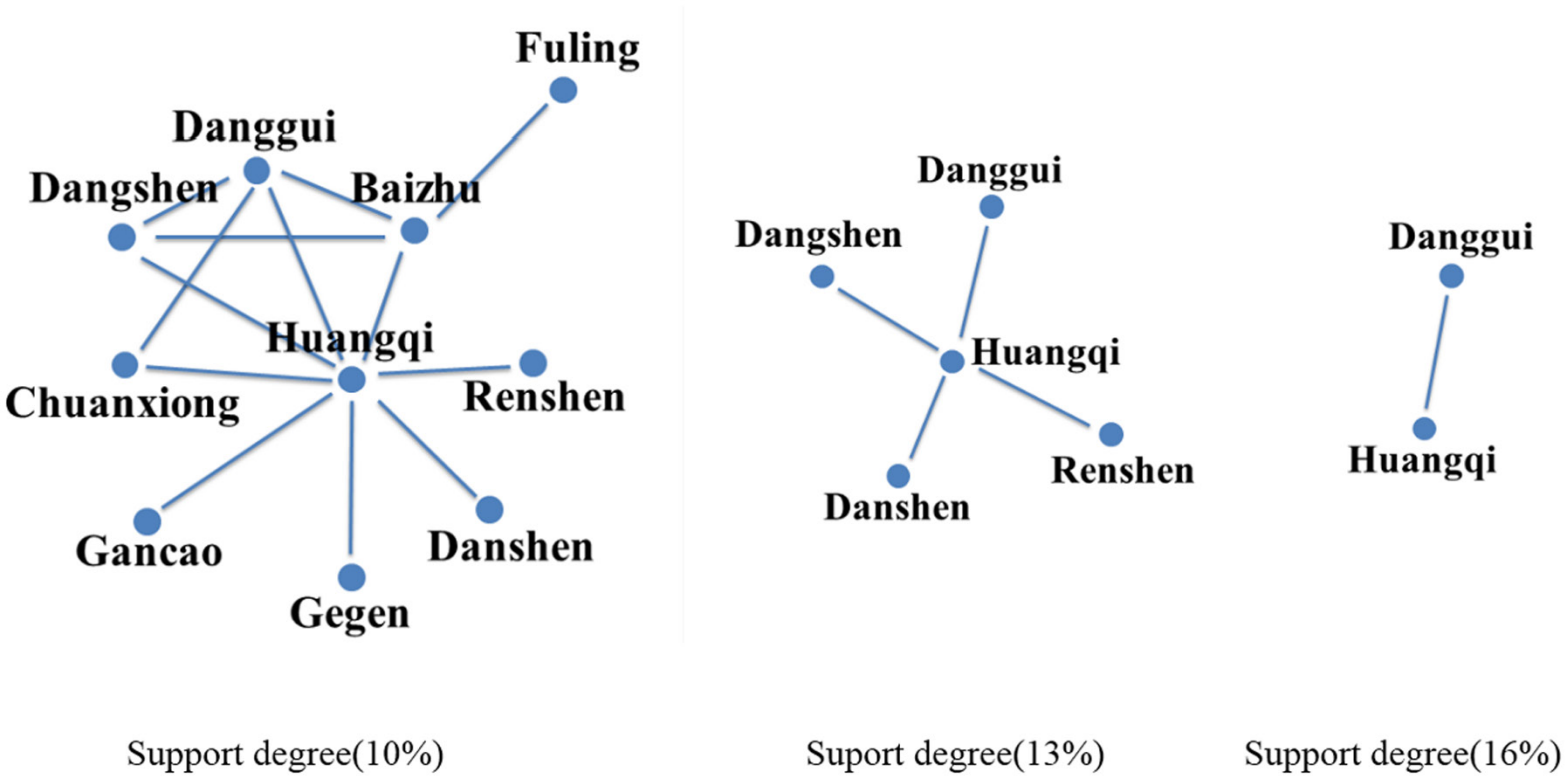
The compatibility of supplement‐Qi TCMs.

The analysis of compatibility patterns in TCM prescriptions for supplementing Qi has identified a core set of compatibility rules. The association network, visualized in Figure 1, illustrates the relationships between herbs with varying degrees of support, set at 10%, 13%, and 16%, respectively. Notably, as the support degree decreases, only the combination of Huangqi and Danggui remains in the compatibility network. This observation underscores the robustness of the Huangqi and Danggui pairing as the most effective combination for supplementing Qi, highlighting its significance in TCM practice for enhancing vital energy and overall health.

### 3.2 The combination of Huangqi and Danggui exerts anti‐tumor effects and improves immunity

**FIGURE 2:**
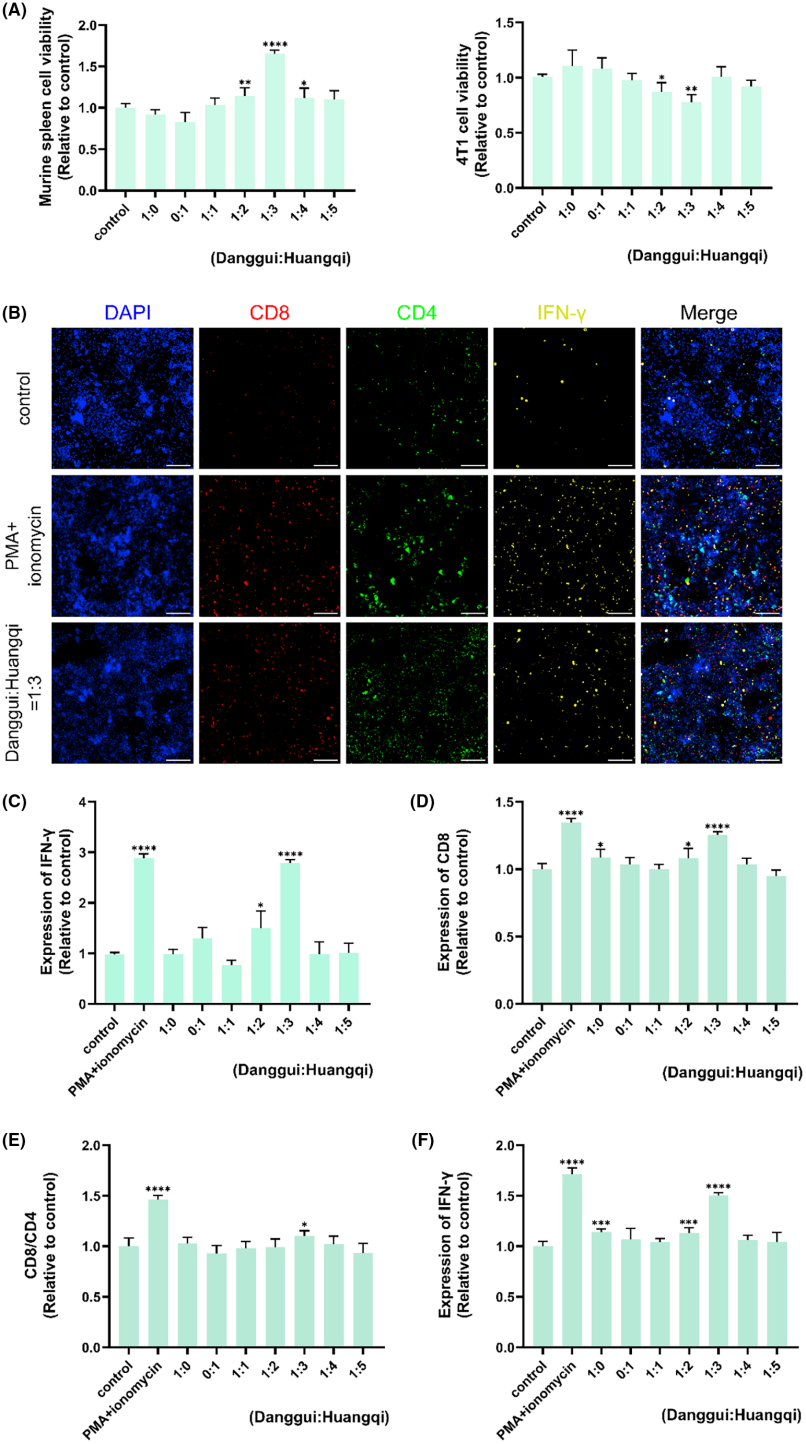
The combination of Danggui and Huangqi has anti‐tumor effects and improves immunity. (A) Effects of different proportions of Danggui and Huangqi on the activity of murine spleen cells and 4T1 cells. (B) The effects of the Danggui and Huangqi combination (1:3) on CD4, CD8, and IFN‐γ expression in co‐culture cells were evaluated using high content screening. (C) The level of IFN‐γ was tested in co‐culture supernatant using ELISA after treatment with combined Danggui and Huangqi (1:3). (D–F) Effects of different proportions of Danggui and Huangqi on the expression of CD4 (D), CD8/CD4 (E), and IFN‐γ (F) in co‐cultured cells. Data shown are the mean ± SEM of three independent experiments. *p < 0.05, **p < 0.01, ***p < 0.001, and ****p < 0.0001, compared with the control group.

Figure 1 demonstrates that Huangqi is an essential and highly regarded TCM for supplementing Qi. Building on this, we proceeded to investigate the compatibility ratios of Huangqi and Danggui, varying the amount of Huangqi while keeping Danggui constant (Danggui:Huangqi ratios of 1:1, 1:2, 1:3, 1:4, and 1:5). Since the effectiveness of supplementing Qi is closely linked to enhancing the host's immune system, the potential anti‐tumor effects of these herbs may be due to their immune‐regulatory properties. We assessed the impact of Huangqi and Danggui combinations on the viability of murine spleen cells and 4T1 cells. PMA + ionomycin served as a positive control. Notably, when the ratio of Danggui to Huangqi was 1:3, spleen cells exhibited the highest activity, and 4T1 breast cancer cells showed the most pronounced inhibition (Figure 2A). Consequently, subsequent studies utilized co‐cultured cells (4T1 cell: murine spleen cell ratio of 1:1).

Chemotherapy‐induced immune activation was characterized by increased expression of CD4^+^ and CD8^+^ T cells and the production of IFN‐γ.^28^ The influence of the Danggui and Huangqi combination (1:3) on the expression of CD4, CD8, and IFN‐γ in co‐cultured cells was then evaluated using high‐content screening. The findings indicated that treatment with combined Danggui and Huangqi (1:3) increased the expression of CD8 and IFN‐γ (Figure 2B). Additionally, the level of IFN‐γ in the co‐culture supernatant was significantly elevated following treatment with the 1:3 combination of Danggui and Huangqi, as determined by ELISA (Figure 2C). Furthermore, high‐content screening was used to evaluate which combination ratio of Danggui and Huangqi most significantly affected the expression of CD4, CD8, and IFN‐γ in co‐cultured cells. Intriguingly, the expressions of CD8, the CD8/CD4 ratio, and IFN‐γ were most notably increased with a Danggui to Huangqi ratio of 1:3 (Figure 2D–F).

Therefore, a ratio of one part Danggui to three parts Huangqi was identified as the optimal combination for anti‐tumor activity through the enhancement of immune function. In subsequent studies, a 1:3 ratio of Danggui to Huangqi was consistently used. All results consistently showed that the combination of Huangqi and Danggui can activate the immune system to suppress tumor growth and is more effective than a single medicinal herb.

### 3.3 Combined Danggui and Huangqi inhibits breast cancer in vivo by activating the immune system

**FIGURE 3:**
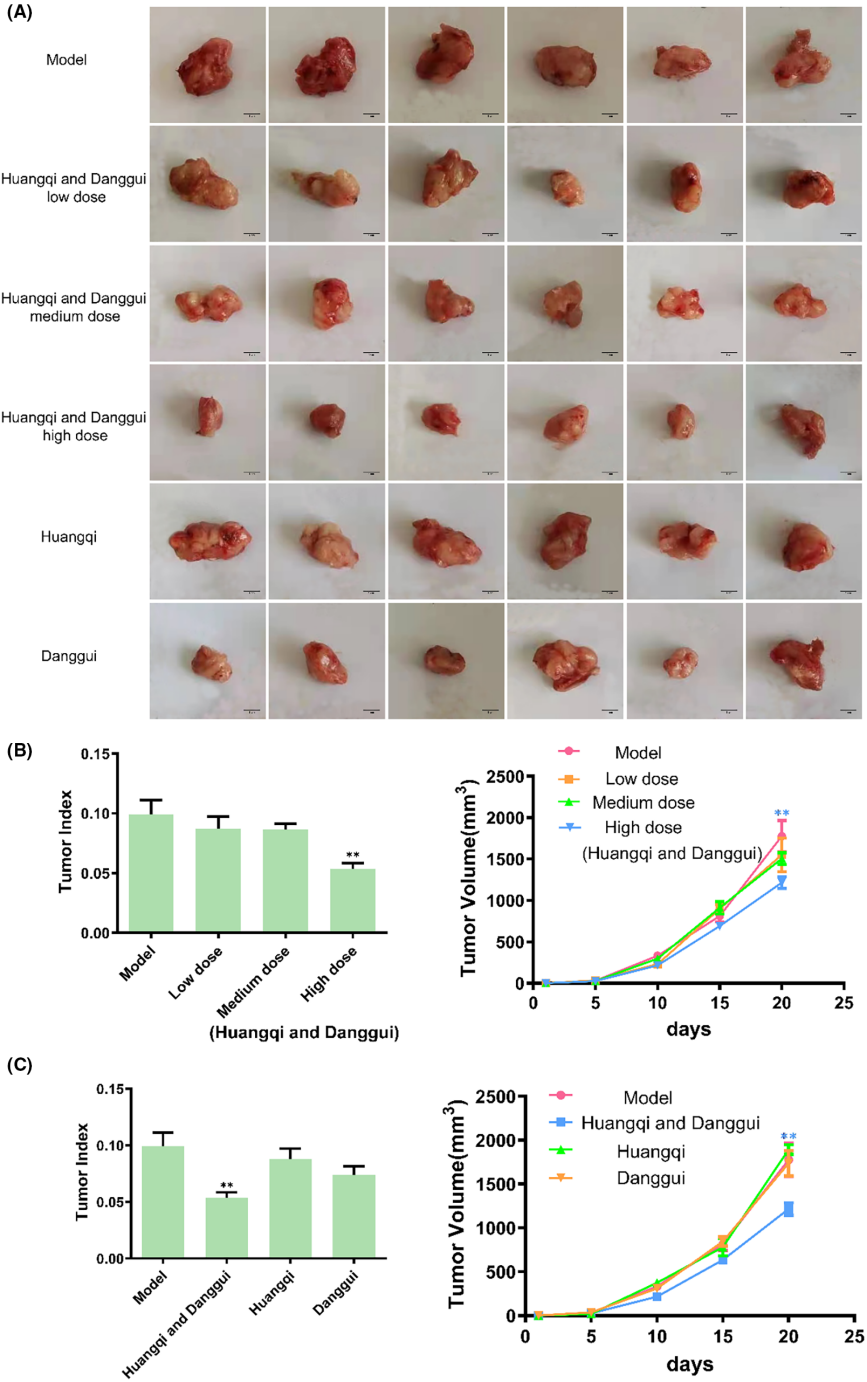
Combined Danggui and Huangqi inhibit breast cancer growth. (A) The therapeutic effects of combined Danggui and Huangqi (1:3) were examined for 4T1 cells xenograft mice. (B) Tumor weight and growth curves were tested after treatment of different doses of combined Danggui and Huangqi (1:3). (C) Tumor index and volume were tested after treatment with a high dose of combined Danggui and Huangqi (1:3) or single drugs. Data shown are the mean ± SEM of three independent experiments. *p < 0.05, and **p < 0.01, compared with the model group.

**FIGURE 4:**
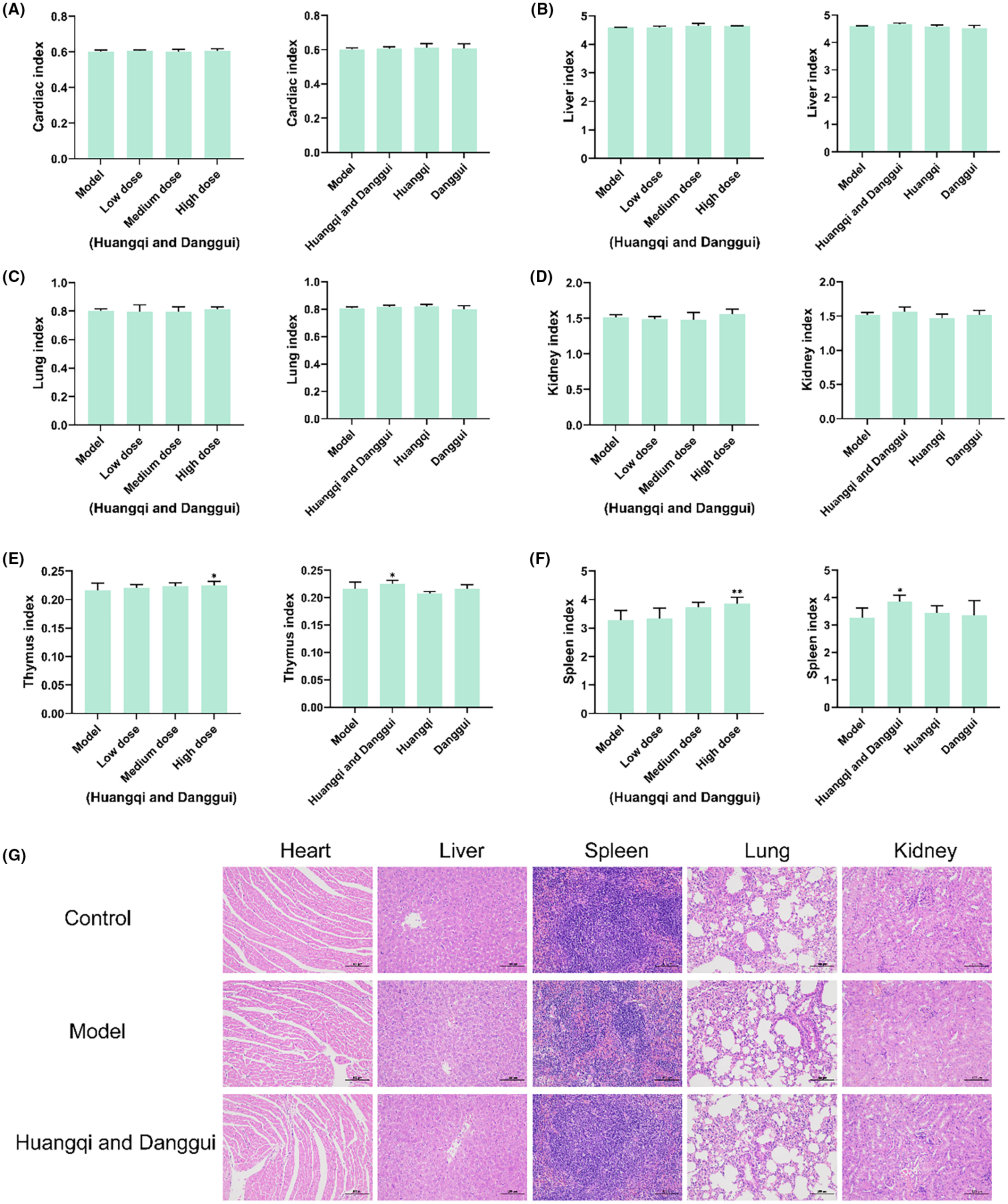
Combined Danggui and Huangqi may inhibit breast cancer growth through immune activation. (A–F) The index of heart (A), liver (B), lung (C), kidney (D), thymus (E), and spleen (F) were tested after treatment with different doses of combined Danggui and Huangqi (1:3) or single drugs. Data shown are the mean ± SEM of three independent experiments. (G) H&E staining of heart, liver, spleen, lung, and kidney after a high dose of combined Danggui and Huangqi. *p < 0.05, and **p < 0.01, compared with the model group.

The therapeutic effects of combined Huangqi and Danggui were examined in vivo. Figure 3A showed the anti‐tumor effects of different doses of combined Huangqi and Danggui as well as single agents. Subsequently, we compared the antitumor effects of different doses of combined Huangqi and Danggui, as well as the antitumor effects of combined Huangqi and Danggui (high‐dose) and single herbs. The findings revealed that a high dose of the combined Huangqi and Danggui significantly reduced tumor growth in 4T1 xenograft mice, and this effect was markedly more potent than that observed with the individual herbs (Figure 3B,C). In addition, we examined the indexes of heart, liver, lung, kidney, thymus, and spleen to evaluate the toxicity of these drugs. Except in spleen, there were no significant changes in heart, liver, lung, kidney, and thymus in all groups (Figure 4A–E). The spleen index was significantly elevated in the high‐dose combined Huangqi and Danggui group compared with the model group (Figure 4F), suggesting that it may inhibit breast cancer growth through immune activation. Moreover, we further evaluated the effect of a high dose of combined Danggui and Huangqi on heart, liver, spleen, lung, and kidney by H&E staining, and the results showed that the combination of Danggui and Huangqi had no toxicity on mice (Figure 4G).

**FIGURE 5:**
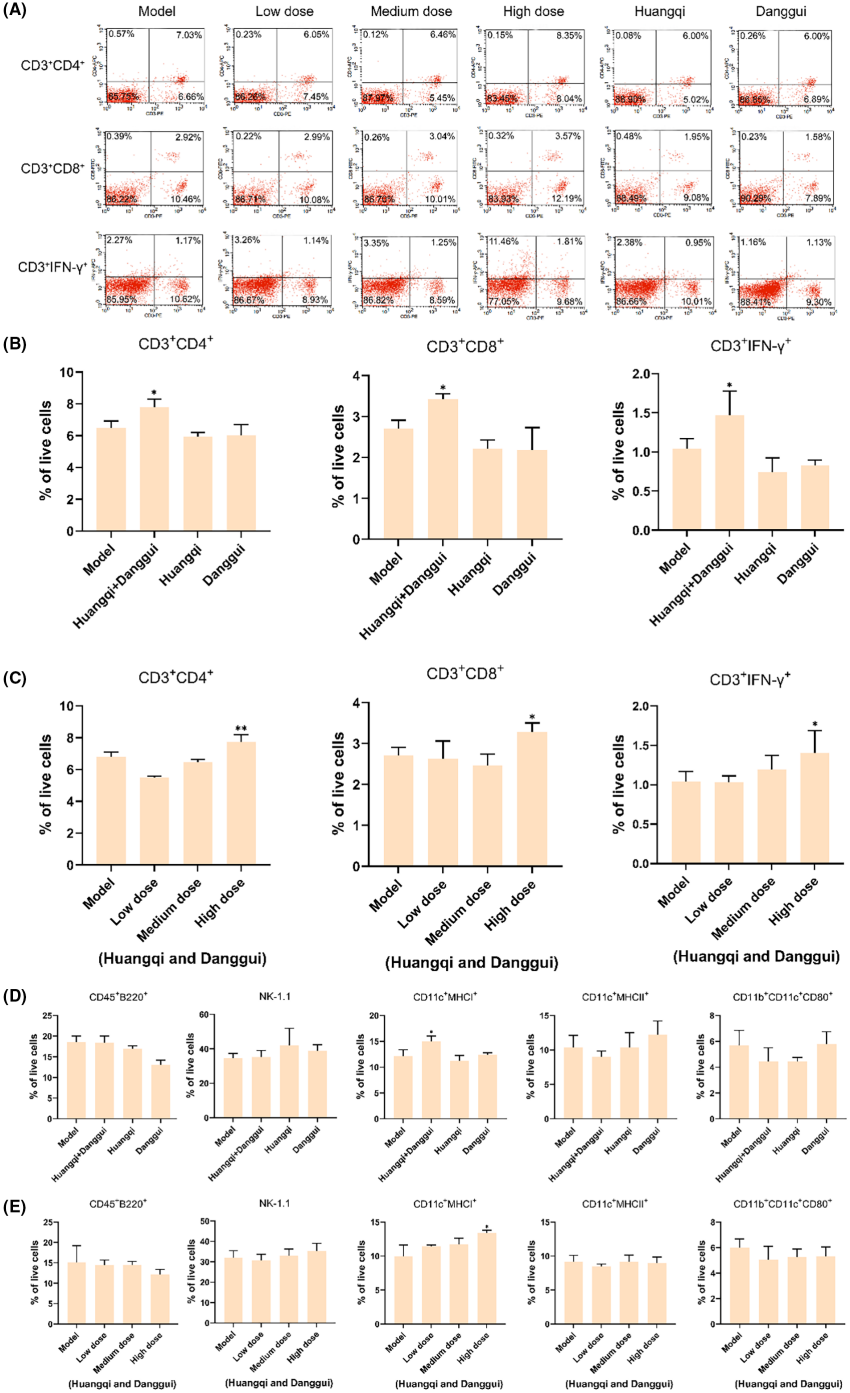
Combined Danggui and Huangqi activate immunity. (A) Flow cytometric analysis of CD3^+^CD4^+^ T cells, CD3^+^CD8^+^ T cells, and CD3^+^IFN‐γ T cells. (B) Flow cytometric analysis of CD3^+^CD4^+^ T cells, CD3^+^CD8^+^ T cells, and CD3^+^IFN‐γ T cells tested after treatment with a high dose of combined Danggui and Huangqi (1:3) or single drugs. (C) Flow cytometric analysis of CD3^+^CD4^+^ T cells, CD3^+^CD8^+^ T cells, and CD3^+^IFN‐γ T cells tested after treatment with a different dose of combined Danggui and Huangqi (1:3). (D) Flow cytometric analysis of CD45^+^B220^+^ cells, NK‐1.1 cells, CD11c^+^MHCI^+^ cells, CD11c^+^MHCII^+^ cells, and CD11b^+^CD11c^+^CD80^+^ cells tested after treatment with a high dose of combined Danggui and Huangqi (1:3) or single drugs. (E) Flow cytometric analysis of CD45^+^B220^+^ cells, NK‐1.1 cells, CD11c^+^MHCI^+^ cells, CD11c^+^MHCII^+^ cells, and CD11b^+^CD11c^+^CD80^+^ cells tested after treatment with different doses of combined Danggui and Huangqi (1:3). Data shown are the mean ± SEM of three independent experiments. *p < 0.05, and **p < 0.01, compared with the model group.

We then further assessed the immune‐stimulating effects of the treatment with Danggui and Huangqi. According to flow cytometric analysis, there was a significant increase in the populations of CD3^+^CD4^+^ T cells, CD3^+^CD8^+^ T cells, and CD3^+^IFN‐γ T cells in the spleen in the high‐dose combined Huangqi and Danggui group when compared to the model group (Figure 5A–C). Additionally, the results indicated a notable upsurge in the numbers of CD11c^+^MHC I^+^ cells within the same high‐dose combined treatment group (Figure 5D,E). However, no significant changes were observed in the counts of B220^+^ cells, NK cells, CD11c^+^MHC II^+^ cells, and CD11b^+^CD11c^+^CD80^+^ cells across the different groups (Figure 5D,E). These findings suggest that the combination of Danggui and Huangqi primarily augments the numbers of killer T cells and suppressor T cells, while also enhancing the expression of IFN‐γ and MHC I, thereby activating tumor immunity. However, this combination does not have a significant impact on B cells, NK cells, and dendritic cells. Consequently, the combined treatment of Huangqi and Danggui exhibits a more potent anti‐tumor effect and a more pronounced activation of the immune system in vivo compared to the individual herbs.

### 3.4 The key target genes and signaling pathways of combined Danggui and Huangqi in anti‐tumor immunity

**FIGURE 6:**
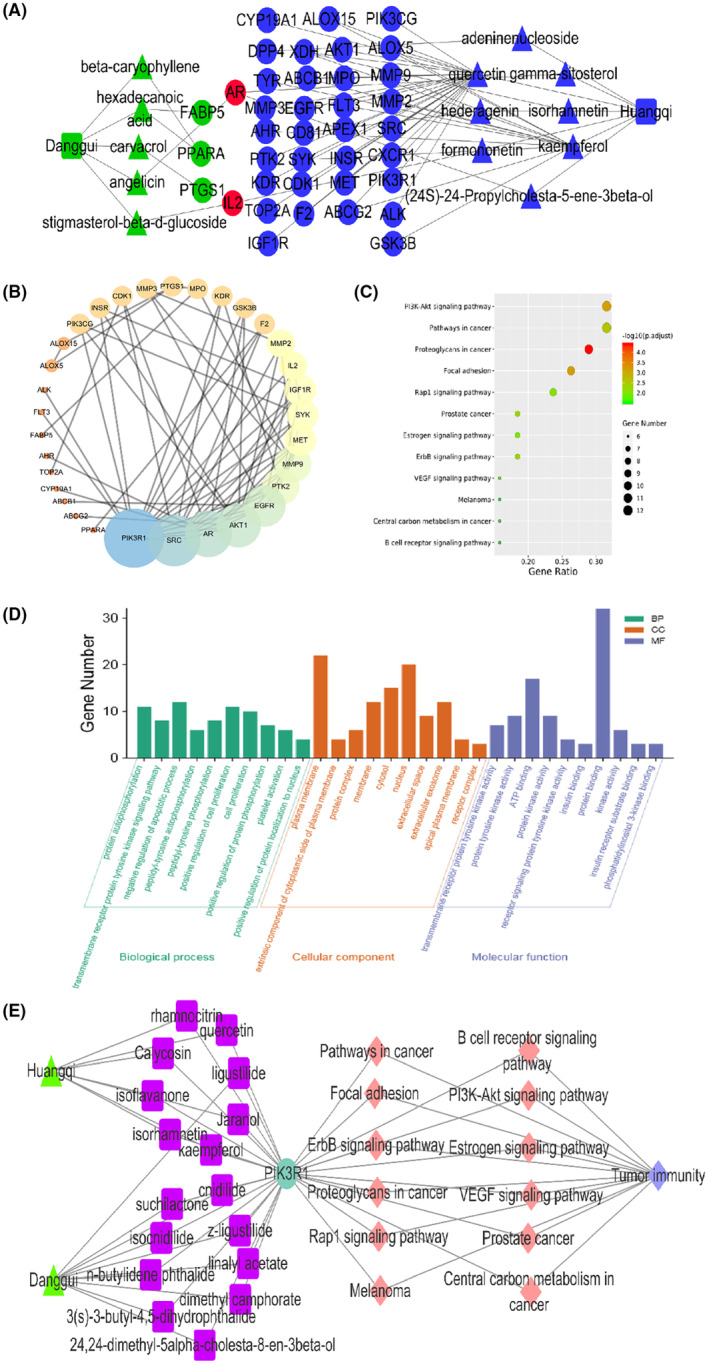
Network pharmacological analysis of combined Danggui and Huangqi. (A) Network analysis of components and gene targets from combined Danggui and Huangqi. (B) The PPI network was established using STRING and Cytoscape. (C) The KEGG pathway enrichment analysis. (D) Biological processes, cellular components, and molecular functions of targeted genes. (E) Cytoscape was used to analyze the network among TCMs, active ingredients, protein targets, the KEGG pathways, and tumor immunity.

Following the removal of duplicate data, a total of 78 major ingredients were identified as potential candidates for Huangqi, while 154 major ingredients were identified for Danggui. After supplementing and eliminating the targets associated with these ingredients, 97 targets related to Huangqi and 29 targets related to Danggui were identified. Utilizing STRING and Cytoscape, a PPI network was established, comprising 32 nodes and 68 edges (Figure 6A,B). Based on the criteria of DC ≥6, BC ≥195.38571, and CC ≥0.5081967, four potential core targets were identified. The PPI network analysis, informed by topological parameters, suggested that *PIK3R1*, *SRC*, *AR*, and *MMP9* could be considered as key target genes for the combined anti‐tumor immunity effects of Danggui and Huangqi (Table 4).

**TABLE 4:** Topological information of 4 potential core targets.

Target	Degree	Betweenness	Closeness
*PIK3R1*	16.0	241.9619	0.5344828
*SRC*	13.0	209.77618	0.56363636
*AR*	11.0	195.38571	0.5344828
*MMP9*	7.0	276.6381	0.5081967

KEGG pathway enrichment analysis identified 12 significant pathways with a *P* value <0.05 and a *Q* value <0.05 (Figure 6C and Table 5). The pathways with the highest degree count values were the PI3K‐Akt signaling pathway (hsa04151, count = 12) and Pathways in cancer (hsa05200, count = 12). Additionally, the molecular function (MF) analysis included PI3K binding (Figure 6D). To provide a systematic and comprehensive explanation of the mechanisms by which the combined use of Huangqi and Danggui enhances tumor immunity, Cytoscape 3.2.1 was employed to analyze the network interactions among TCMs, active ingredients, protein targets, KEGG pathways, and the disease itself. The target *PIK3R1* can be co‐regulated by multiple ingredients to initiate biological effects that are crucial for anti‐tumor immunity (Figure 6E). Significantly, all 12 KEGG pathways listed in Table 5 contain *PIK3R1*, underscoring its potential as the most critical target for the anti‐tumor immune response induced by the combined use of Danggui and Huangqi.

**TABLE 5:** Enrichment analysis on KEGG pathway of potential targets from tumor immunity of Danggui and Huangqi.

Pathway	Gene
hsa05205: Proteoglycans in cancer	*PIK3CG*, *AKT1*, *EGFR*, *IGF1R*, *PTK2*, *MMP9*, *MET*, *MMP2*, ***PIK3R1***, *SRC*, *KDR*
hsa04510: Focal adhesion	*PIK3CG*, *AKT1*, *EGFR*, *IGF1R*, *PTK2*, *GSK3B*, *MET*, ***PIK3R1***, *SRC*, *KDR*
hsa04151: PI3K‐Akt signaling pathway	*PIK3CG*, *AKT1*, *EGFR*, *IGF1R*, *PTK2*, *GSK3B*, *MET*, *INSR*, ***PIK3R1***, *KDR*, *SYK*, *IL2*
hsa05200: Pathways in cancer	*PIK3CG*, *AKT1*, *EGFR*, *IGF1R*, *AR*, *PTK2*, *FLT3*, *GSK3B*, *MMP9*, *MET*, *MMP2*, ***PIK3R1***
hsa04012: ErbB signaling pathway	*PIK3CG*, *AKT1*, *EGFR*, *PTK2*, *GSK3B*, ***PIK3R1***, *SRC*
hsa05215: Prostate cancer	*PIK3CG*, *AKT1*, *EGFR*, *IGF1R*, *AR*, *GSK3B*, ***PIK3R1***
hsa04015: Rap1 signaling pathway	*PIK3CG*, *AKT1*, *EGFR*, *IGF1R*, *MET*, *INSR*, ***PIK3R1***, *SRC*, *KDR*
hsa04915: Estrogen signaling pathway	*PIK3CG*, *AKT1*, *EGFR*, *MMP9*, *MMP2*, ***PIK3R1***, *SRC*
hsa04370: VEGF signaling pathway	*PIK3CG*, *AKT1*, *PTK2*, ***PIK3R1***, *SRC*, *KDR*
hsa05230: Central carbon metabolism in cancer	*PIK3CG*, *AKT1*, *EGFR*, *FLT3*, *MET*, ***PIK3R1***
hsa04662: B cell receptor signaling pathway	*PIK3CG*, *AKT1*, *GSK3B*, *CD81*, ***PIK3R1***, *SYK*
hsa05218: Melanoma	*PIK3CG*, *AKT1*, *EGFR*, *IGF1R*, *MET*, ***PIK3R1***

Bold values PIK3R1 may be the anti‐tumor target of Huangqi and Danggui.

### 3.5 *PIK3R1* is likely a central hub target for the anti‐tumor immunity effects of the combined Danggui and Huangqi

The PPI network and KEGG pathway analyses led us to focus on *PIK3R1* as a key target gene for molecular docking studies. This target was associated with 18 ingredients for the molecular docking process. The docking scores indicating the binding energy for these 18 ingredients were ranked, as detailed in Table 6. And six ingredients with scores above 6 were chosen for further analysis (quercetin, jaranol, isorhamnetin, kaempferol, calycosin, and suchilactone).

**TABLE 6:** Results of molecular docking.

CAS	Name	Score	Structure
1621‐61‐0	Calycosin	8.3685	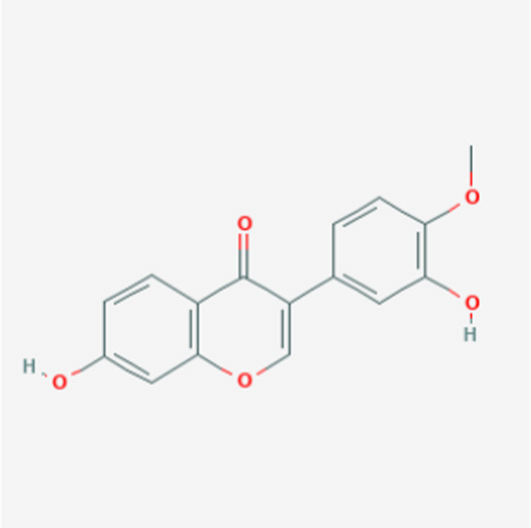
3301‐49‐3	Jaranol	8.2428	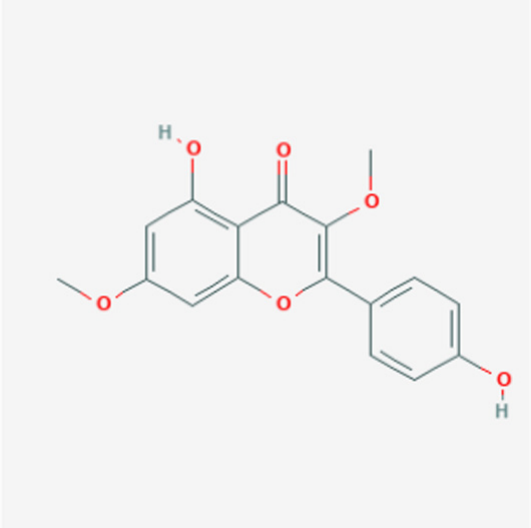
50816‐74‐5	Suchilactone	8.134	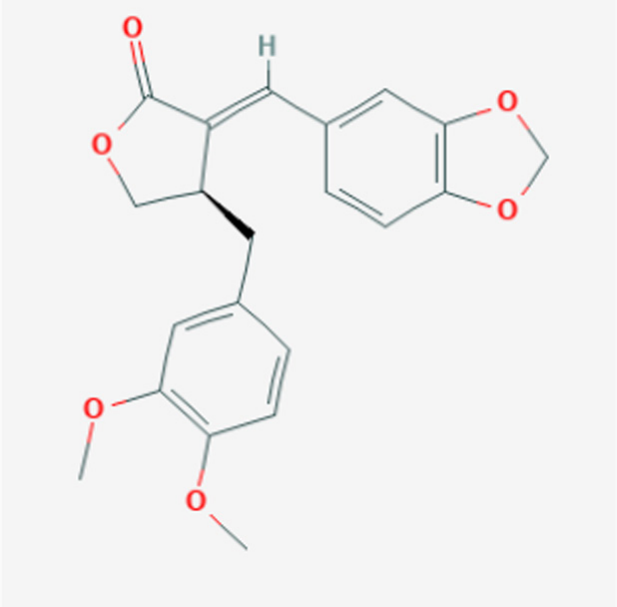
480‐19‐3	Isorhamnetin	7.9825	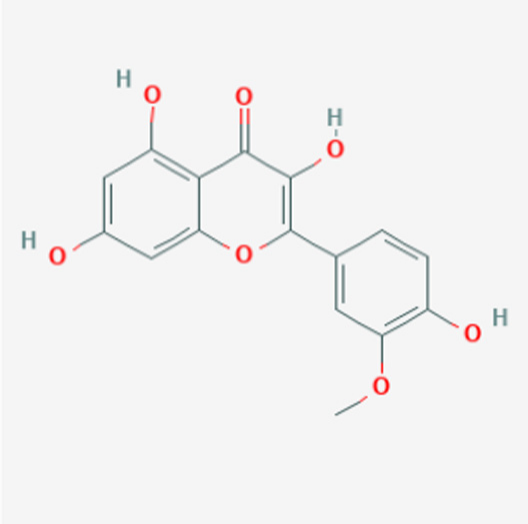
117‐39‐5	Quercetin	6.7683	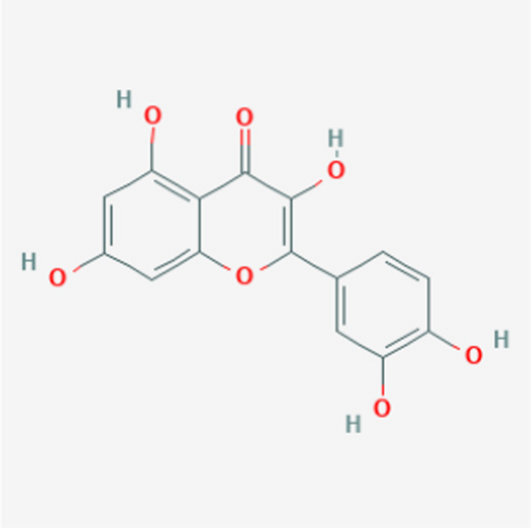
520‐18‐3	Kaempferol	6.0082	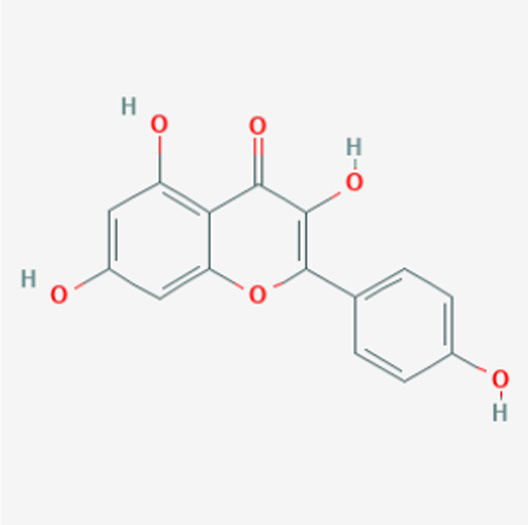
–	3(s)‐3‐butyl‐4,5‐dihydrophthalide	5.8161	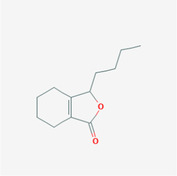
115‐95‐7	Linalyl acetate	5.5716	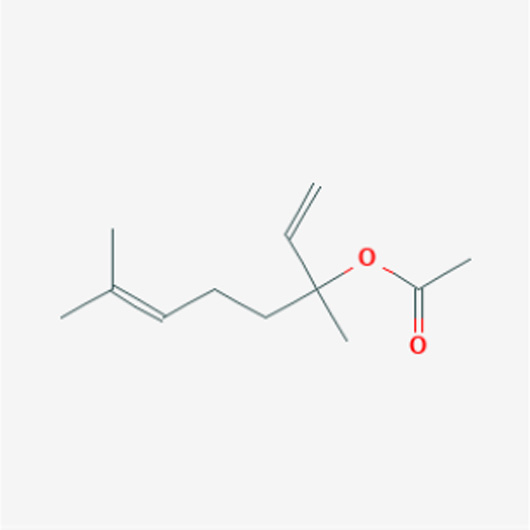
–	24,24‐dimethyl‐5alpha‐cholesta‐8‐en‐3beta‐ol	4.9826	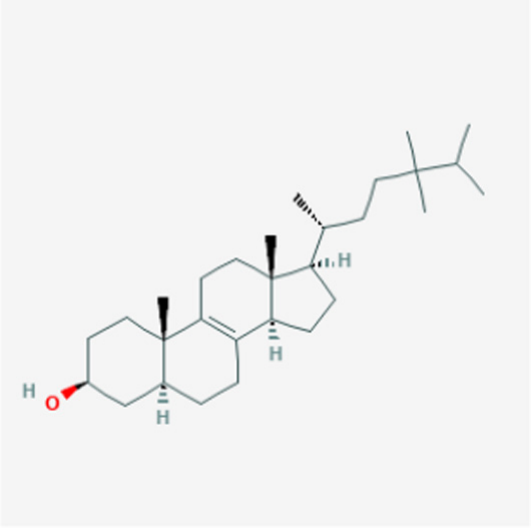
32 383‐76‐9	Medicarpin	4.9571	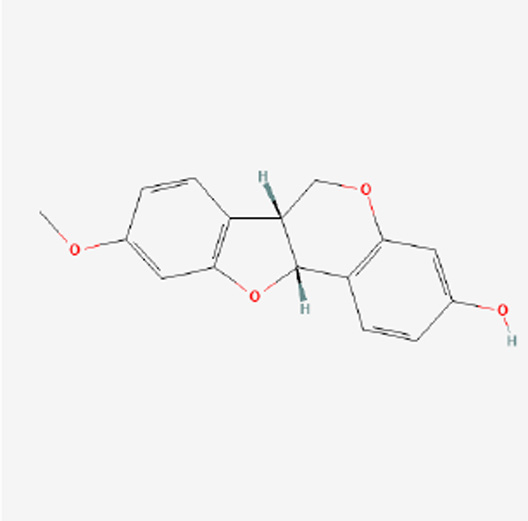
–	n‐butylidene phthalide	4.8898	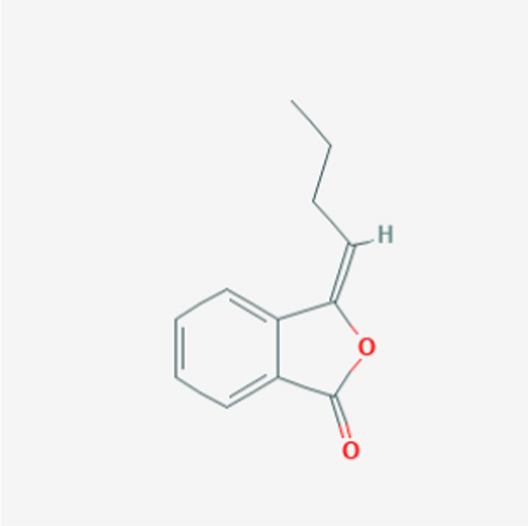
4431‐01‐0	Ligustilide	4.8869	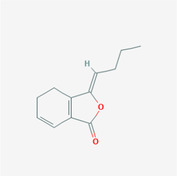
81 944‐09‐4	Z‐ligustilide	4.8869	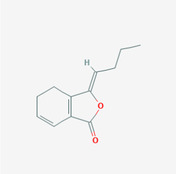
4737‐27‐3	Isoflavanone	4.6343	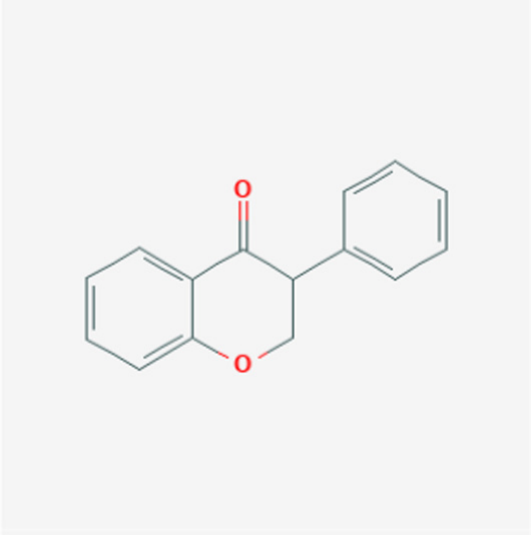
7282‐27‐1	Dimethyl camphorate	4.4653	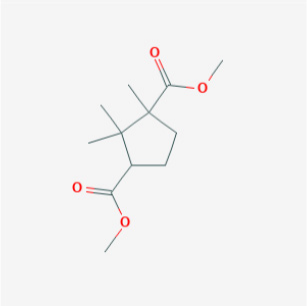
569‐92‐6	Rhamnocitrin	4.2522	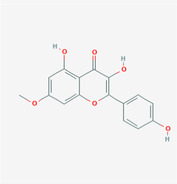
3674‐03‐1	Cnidilide	3.7944	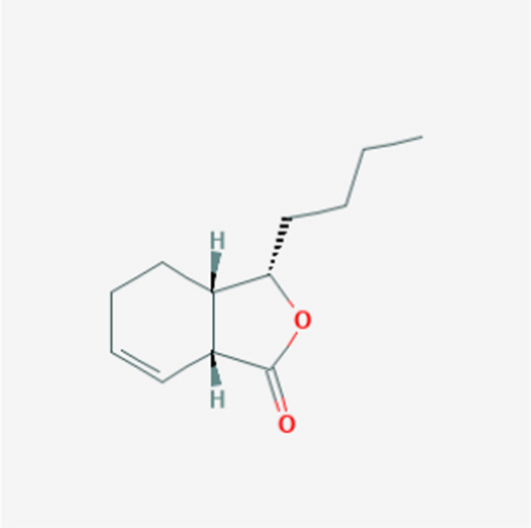
–	Isocnidilide	3.1415	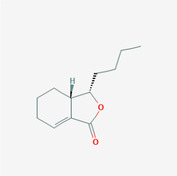

**FIGURE 7:**
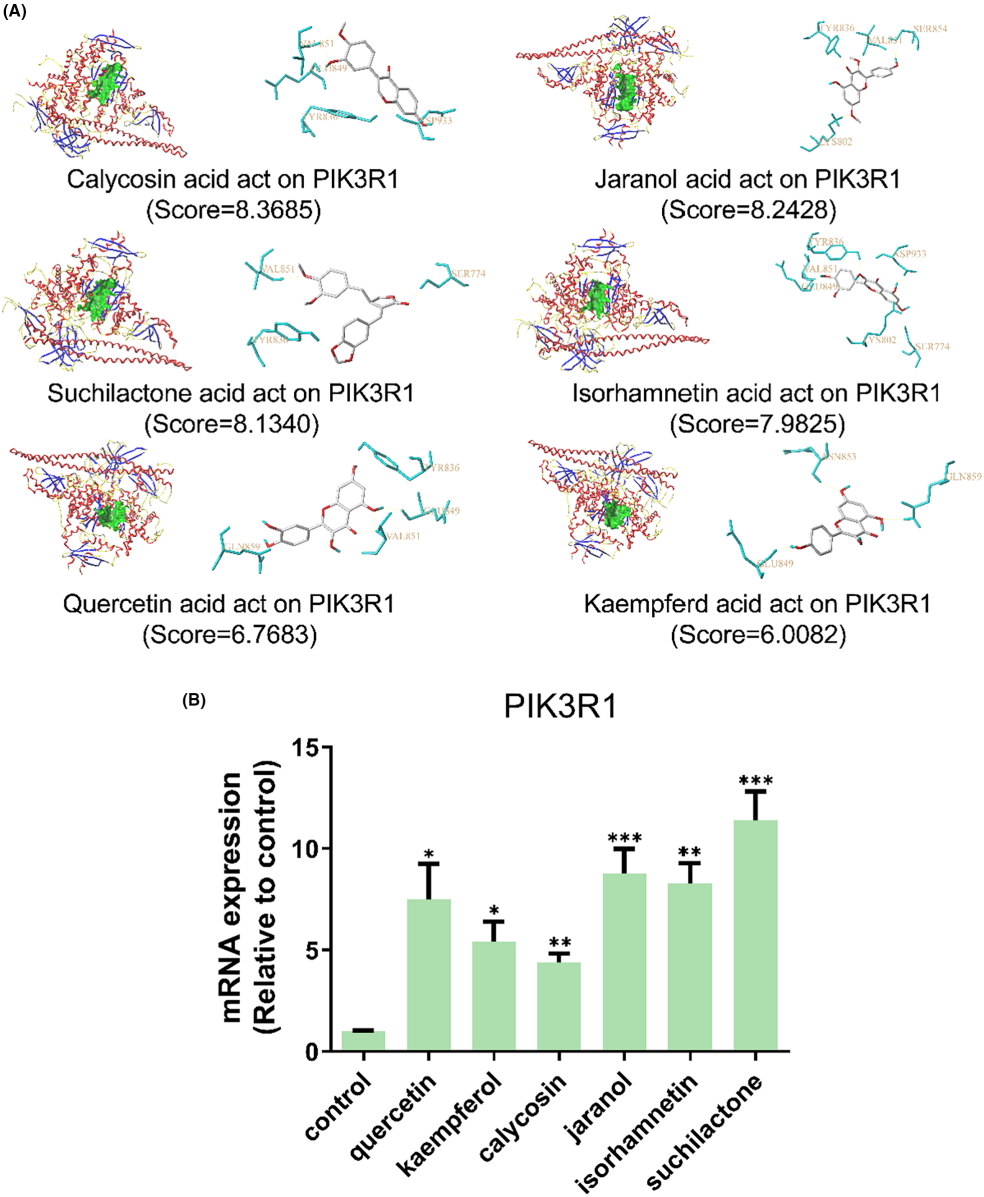
PIK3R1 may be a core hub targets for anti‐tumor immunity of combined Danggui and Huangqi. (A) Molecular docking of PIK3R1 with 6 ingredients (quercetin, jaranol, isorhamnetin, kaempferol, calycosin, and suchilactone). The score indicates the binding energy. (B) The mRNA expression of PIK3R1 was tested in 4T1 cells treated with quercetin, jaranol, isorhamnetin, kaempferol, calycosin, or suchilactone. Data shown are the mean ± SEM of three independent experiments. *p < 0.05, **p < 0.01, and ***p < 0.001, compared with the control group.

As illustrated in Figure 7A, these selected ingredients were found to fit into the active site of the PIK3R1 protein. The six small molecule ingredients can form hydrogen bonds with specific sites on PIK3R1: for quercetin, these are TYR836, GLU849, VAL851, and GLN859; for jaranol, the sites are LYS802, TYR836, VAL851, and SER854; for isorhamnetin, the interacting sites are SER774, LYS802, TYR836, GLU849, VAL851, and ASP933; for kaempferol, the sites are GLU849, ASN853, and GLN859; for calycosin, the sites are TYR836, GLU849, VAL851, and VAL933; and for suchilactone, the sites are SER774, TYR836, and VAL851. Hence, PIK3R1 is a key target of combined Danggui and Huangqi.

The mRNA expression levels of *PIK3R1* in 4T1 cells treated with quercetin, jaranol, isorhamnetin, kaempferol, calycosin, or suchilactone were evaluated. Relative to the control group, the mRNA expression of *PIK3R1* was found to be significantly upregulated in cells treated with these compounds (Figure 7B). These findings suggest that the combined use of Danggui and Huangqi can activate the immune system and thereby suppress the growth of breast cancer cells by targeting the *PIK3R1* gene.

### 3.6 Jaranol is the most important component of combined Danggui and Huangqi, enhancing immunity and inhibiting tumor by targeting *PIK3R1*

**FIGURE 8:**
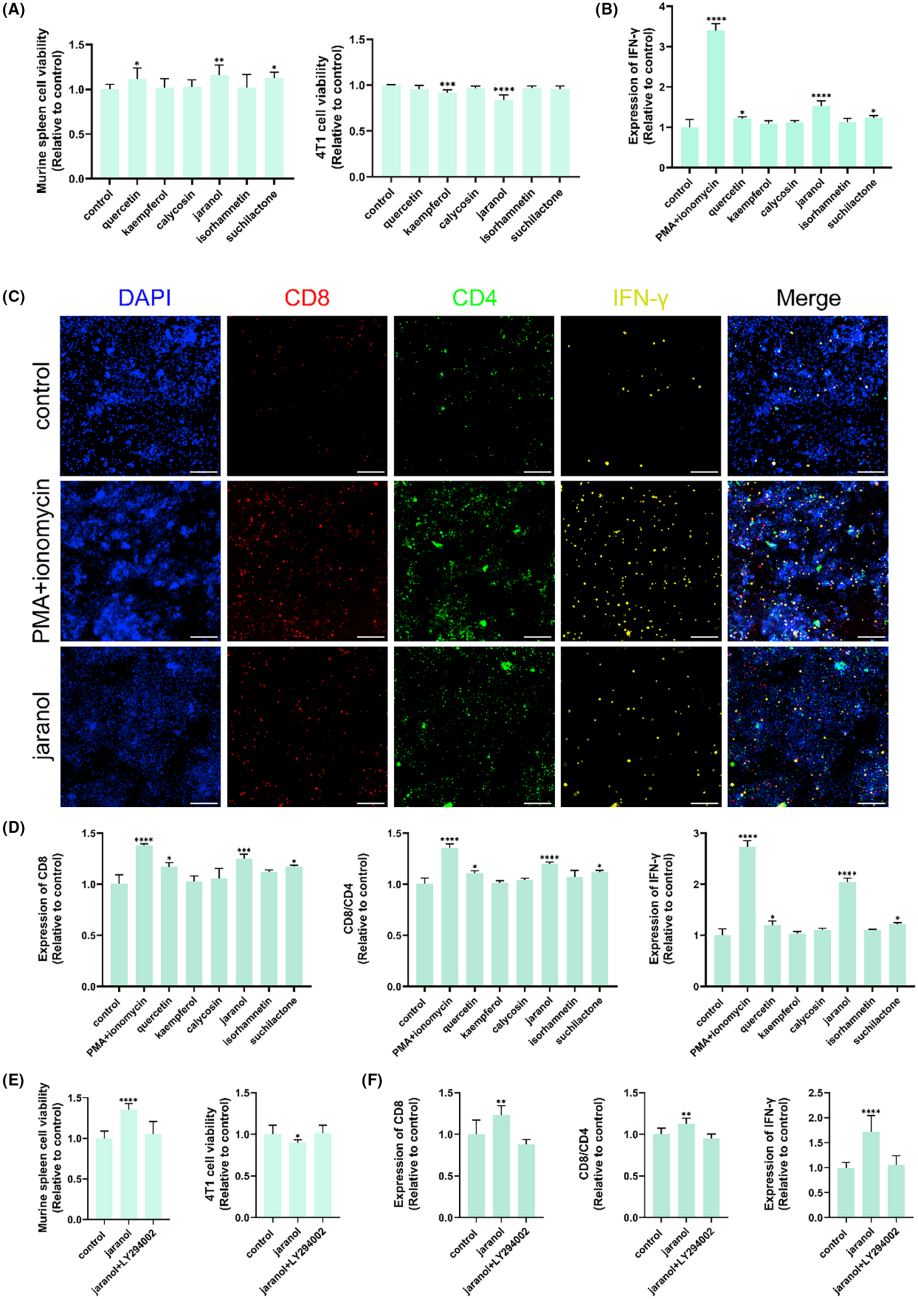
Jaranol is the most important component in the combined Danggui and Huangqi to enhance immunity and inhibit tumor by targeting PIK3R1. (A) Effects of 6 ingredients on the activity of murine spleen cells and 4T1 cells. (B) The level of IFN‐γ was tested in co‐culture supernatant using ELISA after treatment with 6 ingredients. (C) The effects of 6 ingredients on CD4, CD8, and IFN‐γ expression in co‐culture cells were evaluated using high content screening. (D) Effects of 6 ingredients on the expression of CD4, CD8/CD4, and IFN‐γ in co‐culture cells. (E) Effects of jaranol on the activity of murine spleen cells and 4T1 cells were tested after the pretreatment of LY294002. (F) Effects of jaranol on the expression of CD4, CD8/CD4, and IFN‐γ in co‐cultured cells were tested after the pretreatment of LY294002. Data shown are the mean ± SEM of three independent experiments. *p < 0.05, **p < 0.01, ***p < 0.001, and ****p < 0.0001, compared with the control group.

To further clarify the chemical constituents and anti‐tumor mechanisms of the combined therapy with Danggui and Huangqi, six ingredients (quercetin, jaranol, isorhamnetin, kaempferol, calycosin, and suchilactone) were examined for their pharmacological activities. It was observed that quercetin, jaranol, and suchilactone enhanced the viability of murine spleen cells, while kaempferol and jaranol decreased the viability of 4T1 cells (Figure 8A). Additionally, the levels of IFN‐γ, as measured by ELISA, were significantly increased following treatment with quercetin, jaranol, and suchilactone (Figure 8B). The immune activities of these ingredients were further assessed using high‐content screening. Figure 8C illustrates the impact of jaranol on the expression of CD4, CD8, and IFN‐γ. Statistical analysis confirmed that quercetin, jaranol, and suchilactone significantly influenced the expression levels of CD4, the CD8/CD4 ratio, and IFN‐γ (Figure 8D). In conclusion, jaranol not only improves the function of murine spleen cells but also exerts an inhibitory effect on 4T1 cell activity. These findings position jaranol as a potentially key component in the combined use of Danggui and Huangqi, highlighting its role in enhancing immune responses and inhibiting tumor progression.

We subsequently assessed the role of jaranol in anti‐tumor activity and immune system activation, with a focus on its interaction with the target *PIK3R1*. For this, LY294002, a PI3K inhibitor, was employed. Our findings revealed that LY294002 was capable of reversing the splenic cell enhancement effect induced by jaranol, as well as the inhibitory effect on 4T1 cells (Figure 8E). Additionally, the immunomodulatory activity of jaranol was also found to be reversible by LY294002 (Figure 8F). These results collectively indicate that jaranol is a crucial component in the combined Danggui and Huangqi formulation, significantly contributing to the enhancement of immune function and the inhibition of tumor growth through its interaction with *PIK3R1*.

### 3.7 The synergistic combination of suchilactone and kaempferol is pivotal for the anti‐tumor efficacy of the combined therapy with Danggui and Huangqi

**FIGURE 9:**
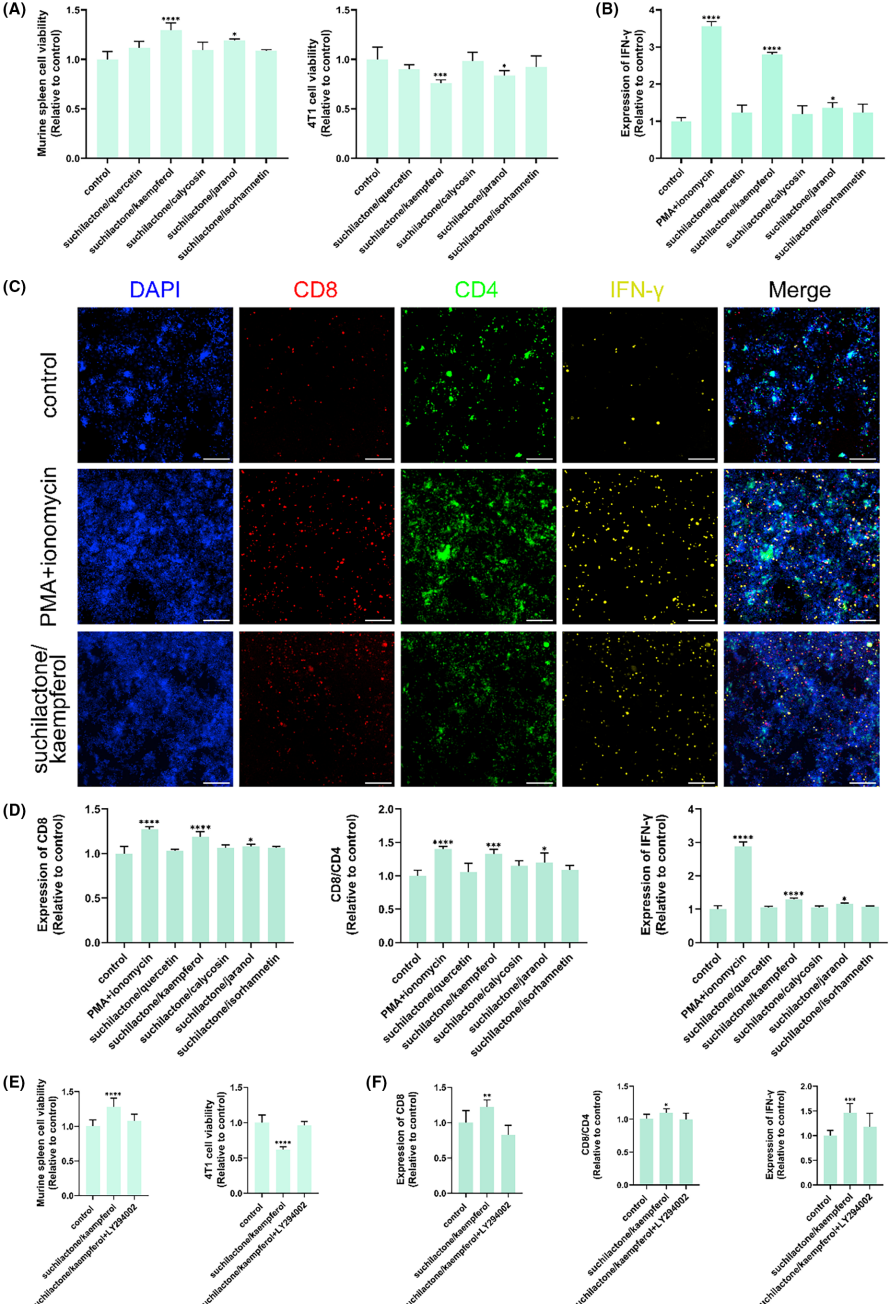
The combination of suchilactone and kaempferol is key for anti‐tumor effect of combined Danggui and Huangqi. (A) Effects of combined suchilactone and the five Huangqi‐derived ingredients (suchilactone/quercetin, suchilactone/kaempferol, suchilactone/calycosin, suchilactone/jaranol, and suchilactone/isorhamnetin) on the activity of murine spleen cells and 4T1 cells. (B) The level of IFN‐γ was tested in co‐culture supernatant using ELISA after the treatment with combined suchilactone and the five Huangqi‐derived ingredients. (C) The effects of combined suchilactone and the five Huangqi‐derived ingredients on CD4, CD8, and IFN‐γ expression in co‐culture cells were evaluated using high content screening. (D) Effects of combined suchilactone and the five Huangqi‐derived ingredients on the expression of CD4, CD8/CD4, and IFN‐γ in co‐cultured cells. (E) Effects of combined suchilactone and the five Huangqi‐derived ingredients on the activity of murine spleen cells and 4T1 cells were tested after the pretreatment of LY294002. (F) Effects of the suchilactone/kaempferol combination on the expression of CD4, CD8/CD4, and IFN‐γ in co‐cultured cells were tested after the pretreatment of LY294002. Data shown are the mean ± SEM of three independent experiments. *p < 0.05, **p < 0.01, ***p < 0.001, and ****p < 0.0001, compared with the control group.

The combination of Danggui and Huangqi has been found to be more effective than the use of a single medicinal herb, and further elucidation of the pharmacodynamic components and underlying mechanisms is warranted. Among the active compounds, quercetin, jaranol, isorhamnetin, kaempferol, and calycosin are derived from Huangqi, while suchilactone is unique to Danggui. Consequently, suchilactone was paired with each of the five Huangqi‐derived ingredients for subsequent experimental investigations. The combinations of suchilactone with kaempferol and suchilactone with jaranol were observed to enhance the activity of murine spleen cells and to inhibit the viability of 4T1 cells (Figure 9A). Both combinations were also found to elevate the secretion levels of IFN‐γ (Figure 9B), as well as increase the expression levels of CD4, the CD8/CD4 ratio, and IFN‐γ (Figure 9D). Notably, the combination of suchilactone with kaempferol demonstrated a more pronounced immune activation and tumor inhibition effect compared to the suchilactone/jaranol combination. The fluorescence expression of CD4, CD8, and IFN‐γ was found to be upregulated by the suchilactone/kaempferol combination, as presented in Figure 9C. These findings suggest that the suchilactone/kaempferol pairing may offer a more potent approach to enhancing immune responses and suppressing tumor growth in the context of combined Danggui and Huangqi therapy.

Similarly, LY294002 was utilized to determine whether the suchilactone/kaempferol combination exerts its role in immune activation and anti‐tumor activity by targeting *PIK3R1*. The findings revealed that the suchilactone/kaempferol combination indeed activates murine spleen cells and exerts inhibitory effects on tumor cell activity through the targeting of *PIK3R1* (Figure 9E). Furthermore, LY294002 was able to reverse the effects of the suchilactone/kaempferol combination on the increased expression of CD4, CD8/CD4, and IFN‐γ (Figure 9F). Thus, the suchilactone/kaempferol combination activates the immune system and inhibits tumor growth by targeting *PIK3R1*, establishing its importance in the anti‐tumor effects of the combined therapy with Danggui and Huangqi.

**FIGURE 10:**
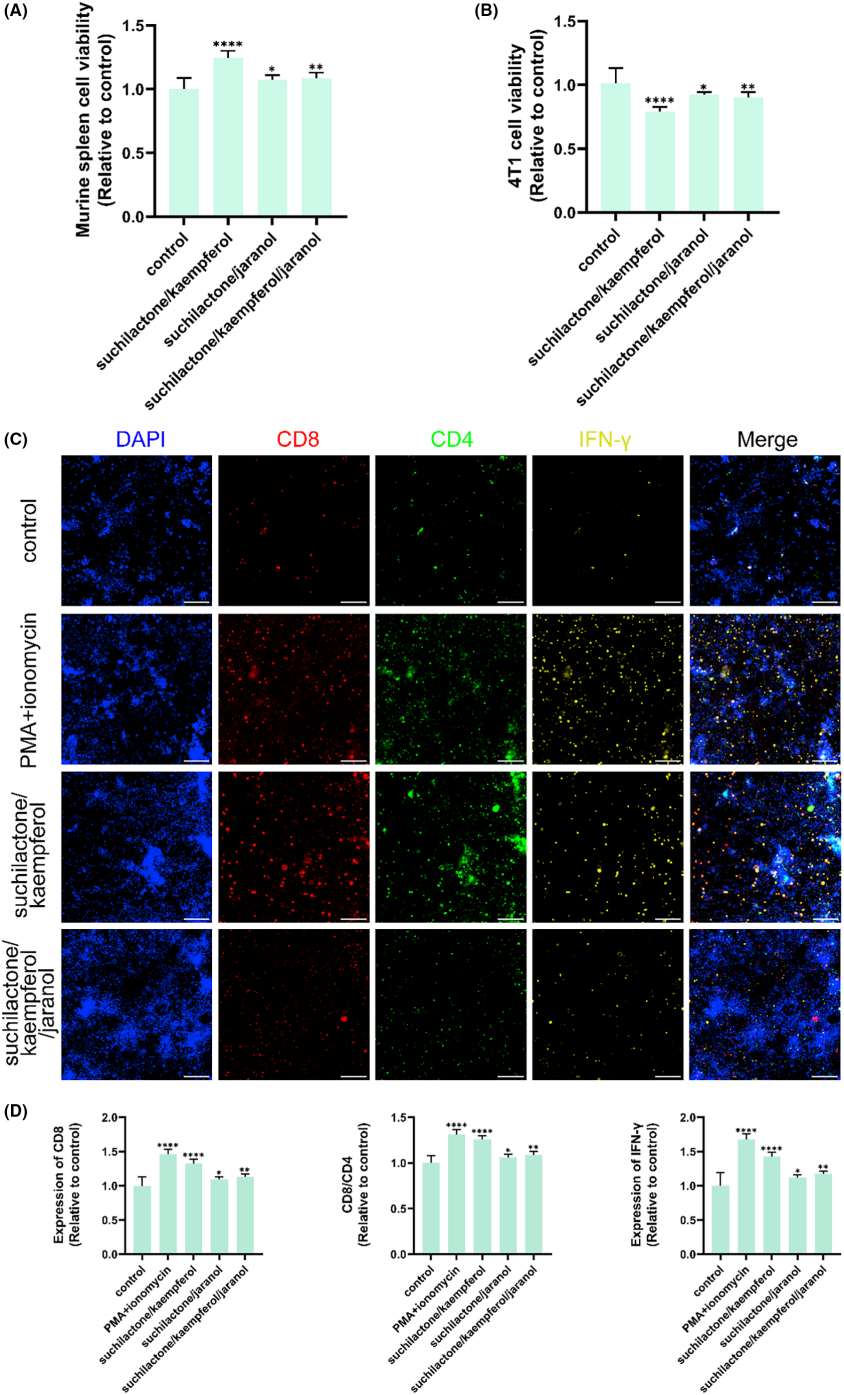
The anti‐tumor effect of combined suchilactone and kaempferol is stronger than the suchilactone/kaempferol/jaranol combination. (A and B) Effects of suchilactone/kaempferol combination, suchilactone/jaranol combination, and suchilactone/kaempferol/jaranol combination on the activity of murine spleen cells (A) and 4T1 cells (B). (C) High content screening detected the expression of CD4, CD8, and IFN‐γ in co‐cultured cells treated by suchilactone/kaempferol combination, suchilactone/jaranol combination, and suchilactone/kaempferol/jaranol combination. (D) Effects of suchilactone/kaempferol combination, suchilactone/jaranol combination, and suchilactone/kaempferol/jaranol combination on the expression of CD4, CD8/CD4, and IFN‐γ in co‐cultured cells. Data shown are the mean ± SEM of three independent experiments. *p < 0.05, **p < 0.01, ***p < 0.001, and ****p < 0.0001, compared with the control group.

Although the combination of suchilactone with kaempferol demonstrated a more enhanced anti‐tumor effect compared to the suchilactone/jaranol combination, these two combinations fully confirmed the significant anti‐tumor effect of the combination of Danggui and Huangqi. And jaranol is the most important component in the combined Danggui and Huangqi to inhibit tumor. Thus, we tested whether the combination of these three ingredients (suchilactone/kaempferol/jaranol) had a stronger anti‐tumor effect. Interestingly, the suchilactone/kaempferol/jaranol combination demonstrated a weaker immune activation and tumor inhibition effect compared to the combination of suchilactone with kaempferol (Figure 10A,B). Figure 10C shows the fluorescence expression of CD4, CD8, and IFN‐γ by the suchilactone/kaempferol combination and the suchilactone/kaempferol/jaranol combination. According to statistical analysis, compared with the suchilactone/kaempferol/jaranol combination, the combined suchilactone and kaempferol had significantly stronger effects on increasing the expression of CD4, CD8/CD4 and IFN‐γ (Figure 10D). Therefore, by activating immunity, the synergistic combination of suchilactone and kaempferol is pivotal for the anti‐tumor efficacy of the combined therapy with Danggui and Huangqi.

## 4 DISCUSSION

In the context of tumor treatment, TCM prioritizes the restoration of bodily balance and the enhancement of host defense mechanisms, which include boosting immunity, as well as employing certain cytotoxic herbal remedies.^29^ The principle of supplementing Qi and bolstering the body's resistance is a fundamental approach in TCM for treating tumors.^30^ Moreover, the supplementation of Qi is recognized for its ability to improve host immunity.^17^, ^18^ Numerous TCM formulas designed to tonify Qi incorporate Huangqi and Danggui. A prime example is the Bu Zhong Yi Qi decoction, a significant Qi‐supplementing formula in TCM that includes both Huangqi and Danggui. Through TICMISS regularity analysis, it has been observed that Huangqi and Danggui form the most frequently used combination for Qi supplementation, with Huangqi being an essential medicinal herb for this purpose.

Out of the six components identified as targeting *PIK3R1*, five originate from the Huangqi herb, with jaranol being the most critical component in the combined use of Danggui and Huangqi to enhance immunity and inhibit tumor growth by interacting with *PIK3R1*. It is important to note that jaranol is exclusive to Huangqi, while suchilactone is derived solely from Danggui. Furthermore, the combinations of suchilactone with kaempferol, as well as suchilactone with jaranol, have been shown to enhance the activity of murine spleen cells and to suppress the viability of 4T1 cells, indicating a higher proportionate requirement for Huangqi in the mixture. However, research has indicated that an overactivated immune system can inadvertently provide tumor cells with signals for growth and create a conducive environment for their proliferation.^31^, ^32^ Consequently, a balance must be struck to optimize the therapeutic benefits. Findings suggest that when the ratio of Danggui to Huangqi is 1:3, spleen cells exhibit the most robust activity, and breast cancer cells experience the most pronounced inhibitory effects. This ratio potentially offers the most effective therapeutic strategy for clinical applications, aligning with the TCM approach to cancer treatment that emphasizes a balanced and holistic method of restoring health and combating disease.

PIK3R1 is recognized as a regulatory subunit of class IA phosphoinositide 3‐kinases (PI3Ks), interacting with, stabilizing, and inhibiting the catalytic subunits PI3K‐p110.^33^ Its expression profile varies across different types of human tumors. For instance, PIK3R1 exerts an inhibitory function in hepatocellular carcinoma and renal cancer,^33^, ^34^ while it acts as an oncogene in ovarian and colon cancers, contributing to tumor growth and metastasis.^35^, ^36^ Recent studies have indicated that mutations or deficiencies in PIK3R1 can impair immune regulation and immune cell function,^37^ highlighting its intimate connection with the immune system. It has been observed that PIK3R1 expression is reduced in CD8^+^ T cells,^38^ and PIK3R1 deficiency has been identified as a novel resistance mechanism to pharmacological Notch inhibition in patients with refractory or relapsed T‐cell acute lymphoblastic leukemia.^39^ Our network pharmacology analysis suggests that Huangqi and Danggui modulate the expression of PIK3R1, thereby enhancing immunity and inhibiting tumor growth. It is plausible that Huangqi and Danggui activate tumor immunity by upregulating the expression of PIK3R1 in T cells, which could play a critical role in the inhibition of breast cancer. Molecular docking studies have revealed the interaction of PIK3R1 with compounds such as quercetin, jaranol, isorhamnetin, kaempferol, and calycosin from Huangqi, as well as suchilactone from Danggui. Among these, jaranol emerges as the most significant component in the combined use of Danggui and Huangqi for enhancing immunity and inhibiting tumor growth by upregulating *PIK3R1*. To date, there have been no reports on the anti‐tumor effects of jaranol, indicating a potential area for further research and investigation. Understanding the precise role of these compounds and their mechanisms of action could pave the way for the development of novel therapeutic strategies that leverage the immune system's capabilities in the fight against cancer.

The synergistic combination of Danggui and Huangqi has been found to be more effective than the use of a single medicinal component. Among the six ingredients identified to bind with the target PIK3R1, suchilactone is unique to Danggui. When combined with the other five ingredients originating from Huangqi, suchilactone helps to elucidate the active constituents of the combined Danggui and Huangqi formulation. Specifically, the combinations of suchilactone with kaempferol and suchilactone with jaranol have been shown to enhance immune response and suppress tumor growth by targeting *PIK3R1*. Importantly, jaranol is the most important component in the combined Danggui and Huangqi to inhibit tumor, while kaempferol does not show a significant effect in this context. However, compared with the suchilactone/jaranol combination, the combination of suchilactone with kaempferol demonstrated a more enhanced anti‐tumor effect. Interestingly, when three ingredients (suchilactone/kaempferol/jaranol) were combined, their anti‐tumor effect was weaker than that of the combined suchilactone and kaempferol. It is worth mentioning that kaempferol, a natural flavonoid abundant in various vegetables, fruits, and TCMs, has been reported to possess anti‐tumor activity against a range of cancers, including gastric, lung, and renal cancer,^40^, ^41^ so kaempferol may play a vital role in the compatibility of Danggui and Huangqi. These results revealed that the synergistic combination of suchilactone and kaempferol is pivotal for the anti‐tumor efficacy of the combined therapy with Danggui and Huangqi by activating immunity.

These findings suggest that the use of drug combinations could be a more effective strategy for future chemotherapy approaches in treating diseases, particularly by leveraging the immune system's capabilities to combat cancer. The synergistic interactions between different components in a formulation lead to enhanced therapeutic outcomes that may not be achievable with single‐agent treatments. This underscores the importance of exploring and optimizing herbal combinations in TCM to maximize their potential benefits in clinical applications.

## 5 CONCLUSIONS

In conclusion, our study presents a thorough and methodical examination of the combined use of Huangqi and Danggui as a potential anti‐tumor therapy. The results of our research indicate that this herbal combination can activate the immune system to suppress tumor growth, proving to be more efficacious than individual herbs in vitro and in vivo. The enhancement of immune function through the targeting of *PIK3R1* is a significant mechanism by which this combination exerts its anti‐breast cancer effects. Thus, the synergistic combination of Huangqi and Danggui holds promise as an effective adjuvant therapy for breast cancer, with potential applications in clinical practice. This approach not only leverages the inherent strengths of TCM but also integrates them with the modern understanding of cancer treatment, offering a new avenue for therapeutic strategies that could benefit patients with breast cancer.

## AUTHOR CONTRIBUTIONS

Hai‐Xin Liu and Qing‐Shan Li conceived and designed the project. Hai‐Xin Liu, Li Lian, and Cai‐Xia Liu performed the experiments. Shi‐Yuan Wen participated in the scientific discussion and research design. Shi‐Yuan Wen and Hai‐Xin Liu wrote and revised the manuscript.

## FUNDING INFORMATION

This work was supported by Natural Science Foundation of Shanxi Province for Youths (No. 20210302123310, No. 20210302124668), Science and technology innovation ability cultivation program project of Shanxi University of Chinese Medicine (No. 2022PY‐TH‐17), and the immune regulation Chinese medicine research and development innovation team project (No. 2022TD1017).

## CONFLICT OF INTEREST STATEMENT

The authors declare no conflicts of interest.

## ETHICS STATEMENT

All animal experiments were conducted in accordance with the principles of good laboratory animal care and performed in compliance with the Animal Ethics Review Committee of Shanxi University of Chinese Medicine.
